# Deep brain stimulation versus motor cortex stimulation for neuropathic pain: A minireview of the literature and proposal for future research

**DOI:** 10.1016/j.csbj.2016.06.003

**Published:** 2016-06-16

**Authors:** C. Michael Honey, Volker M. Tronnier, Christopher R. Honey

**Affiliations:** a Section of Neurosurgery, University of Manitoba, Winnipeg, Canada; b Department of Neurosurgery, Medical Faculty Lübeck, University Hospital of Schleswig-Holstein, Campus Lübeck, Lübeck, Germany; c Division of Neurosurgery, University of British Columbia, Vancouver, Canada

**Keywords:** Deep brain stimulation, Motor cortex stimulation, Neuropathic pain, Review

## Abstract

The treatment of neuropathic pain remains a public health concern. A growing cohort of patients is plagued by medically refractory, unrelenting severe neuropathic pain that ruins their quality of life and productivity. For this group, neurosurgery can offer two different kinds of neuromodulation that may help: deep brain simulation (DBS) and motor cortex stimulation (MCS). Unfortunately, there is no consensus on how to perform these procedures, which stimulation parameters to select, how to measure success, and which patients may benefit. This brief review highlights the literature supporting each technique and attempts to provide some comparisons and contrasts between DBS and MCS for the treatment of neuropathic pain. Finally, we highlight the current unanswered questions in the field and suggest future research strategies that may advance the care of our patients with neuropathic pain.

## 1 Introduction

Neuropathic pain remains a public health concern. The neurosurgical treatment of neuropathic pain has been hindered by i) ambiguity in its diagnosis, ii) small experiences in many different centres, iii) lack of evidence based guidelines, and iv) and the fact that it can be very difficult to treat. This brief review was invited by the organizing committee of the 2nd International Conference on Deep Brain Stimulation held in Dusseldorf March 15–16, 2016 and was designed to summarize and compare the relevant literature supporting deep brain stimulation (DBS) and motor cortex stimulation (MCS) for treating neuropathic pain. Ultimately, there are no prospective, randomized, controlled trials comparing DBS and MCS for neuropathic pain — so the reader will be left, once again, to choose what they think is best for their patients. This review may provide some guidance for that choice.

This paper will provide a brief background for this discussion and then will summarize the literature supporting DBS and MCS and finally draw some comparisons between the two techniques.

## 2 Background

Neuropathic pain has been defined as “pain arising as direct consequence of a lesion or disease affecting the somatosensory system.” [1] There are many different kinds of neuropathic pain and no reason to believe that one procedure will be superior (or even successful) for all conditions. Indeed, this is the first serious problem with our current literature. Many studies have ‘lumped’ patients with different types of neuropathic pain together when discussing outcomes. This increases the number of patients treated but dilutes the results for any one condition. It is quite clear that the neuropathic pain in an amputated limb is very different from the pain in a limb that has been amputated following a plexus avulsion. Although the patients may look the same with an amputated limb and may describe their pain similarly as the worst pain imaginable, constant and burning, the neurophysiology underpinning their pain is different (peripheral nerve versus spinal cord injury) and their response to neuromodulation appears to be different (better response following peripheral nerve injury). Because there are so many different kinds of neuropathic pain, it is not uncommon for any one neurosurgical centre to have experience with only a few patients of each subtype. This is the second serious problem with our current literature. Only a few centres have a large experience with a single, well-defined subtype of pain. This review will try to categorize the response to DBS or MCS based on pain subtype rather than make sweeping conclusions concerning ‘neuropathic pain’.

Deep brain stimulation has been used for neuropathic pain for over 50 years and similarly, motor cortex stimulation has been used for over 30 years. Despite this long time, there is no consensus on how these operations should be performed. Each neurosurgical centre may have unique differences in surgical technique that could profoundly alter outcome. Most obviously, DBS has been used to stimulate a variety of brain targets for pain relief including the following: septal area (of historical significance and not included in this review), sensory thalamus (the majority of reports), periaqueductal grey or periventricular area, and more recently the anterior cingulate cortex. What is less obvious but equally important is there are no guidelines on post-operative stimulation parameters. It has recently become clear to our group that slight differences in stimulation parameters following MCS can have profound effects on pain outcome [2]. This is the third serious problem with our current literature. There is no consensus on operative technique or post-operative stimulation parameters.

Finally, there is no agreement on how we should evaluate these techniques. If an operation completely alleviates pain in 25% of patients, is it ‘successful’ or a ‘failure’. To an investigator evaluating the technique it may seem an unreliable failure but to the small cohort of patients whose unrelenting pain is gone — it is a blessing. This is the fourth problem with our current literature, we have not agreed how to evaluate these operations. Is any improvement worthwhile or is a 50% pain reduction in at least half the patients required to be deemed successful?

## 3 Deep brain stimulation

DBS was first reported for the treatment of nociceptive pain in 1954 [3]. By the 1970s, many specialized centres were using DBS to treat neuropathic pain. Despite its widespread use for neuropathic pain, only a dozen large series have been published [4]. In the last decade, only eight series with more than six patients have been published. During all that time, only two randomized controlled studies were published. The first, by Marchand et al. [5], reported that placebo stimulation improved pain intensity in a blinded setting whereas thalamic stimulation did not. The second, by Fontaine et al. [6], dealt with cluster headache not neuropathic pain.

The early excitement supporting DBS for pain was dampened by two industry-supported, open label studies that failed to reach their defined targets for success [7]. The first trial, sponsored by Medtronic, was powered to demonstrate that at least half of patients *internalized* would get 50% pain relief. Between 1989 and 1993, 196 patients were enrolled. Those who did not get initial benefit from the implanted electrode during a trial period were not included because their implantable neural stimulator (INS) was not internalized. The trial failed to reach outcome. A second trial, also sponsored by Medtronic, began in 1992 but failed because of lack of accrual by 1998. Without prospective data to support DBS for pain, the Food and Drug Administration in the United States has not allowed its use except as an ‘off label’ device [7].

The few large, open label trials reporting on the outcome of DBS for pain have all suggested the technique is beneficial. Rasche et al. reported 56 patients treated for a variety of neuropathic pain syndromes with a follow up of between 1 to 8 years [8]. Electrodes were implanted in the sensory thalamus and the periventricular grey region and evaluated alone or in combination in a blinded fashion prior to implantation of the stimulator. The best long-term results were obtained in patients with failed back surgery syndrome (FBSS) and neuropathic pain of peripheral origin (also known as CRPS-II). Poor results were seen following central pain due to spinal cord injury or post-stroke pain. In the CRPS-II group, four out of six patients had more than a 50% reduction in pain.

Boccard et al. reported the long-term outcome of 59 patients with DBS in the sensory thalamus, periventricular grey, or both for a variety of neuropathic conditions [9]. After a mean follow-up of almost 20 months, pain was compared to pre-operative levels ‘using a general linear mixed model’. For patients with phantom limb 8/9 improved; for brachial plexus injury 3/6 improved; for post-stroke pain 16/23 improved; for spinal cord injury 4/7 improved; and for cephalalgia 6/11 improved. Improvement was defined as a global improvement of their EuroQol-5D (for the patients that improved, pain reduced by 50% on a visual analogue scale).

Kumar et al. reported the outcome of DBS in the periventricular/periaqueductal grey area (n = 49) or sensory thalamus/internal capsule (n = 16) for a variety of neuropathic pain syndromes [10]. Patients were followed for at least 6 months, mean follow-up was 78 months and success defined as greater than 50% reduction in visual analogue pain scores. For the patients with FBSS, 32/43 had long-term improvement; for peripheral neuropathy 3/5 improved; for thalamic pain 1/5 improved; for trigeminal neuropathy 4/4 improved; for spinal cord injury 0/3; for post-herpetic neuralgia 0/3; and for phantom limb pain 1/1 improved.

These studies have some consistent findings. First, DBS is effective for FBSS. This indication should be reserved for patients who have failed the less invasive spinal cord stimulation (as was the protocol for the trial by Rasche et al). Second, DBS is effective for neuropathic pain of peripheral origin. Third, DBS is poor (but not universally ineffective) for the treatment of pain following spinal cord injury or stroke. Fourth, initial benefit may be lost after several years.

## 4 Motor cortex stimulation

Since its introduction by Tsubakawa in 1991, Motor Cortex Stimulation (MCS) has been used for a variety of neuropathic pain syndromes [11]. Initially used for thalamic pain, it has been tried for many treatment-resistant pain syndromes such as phantom limb pain, postherpetic neuralgia, brachial plexus avulsion, poststroke pain, Wallenberg syndrome, complex regional pain syndrome, pain secondary to multiple sclerosis, spinal cord injury pain, and posttraumatic brain injury pain [12].

Fontaine et al. summarized the literature up to 2006. In summary, a greater than 40% improvement in pain scores were reported in 54% of 117 patients with central pain and 68% of 44 patients with trigeminal neuropathic pain [13]. A recurrent theme appears that MCS is effective for deafferentation pain due to peripheral (phantom limb or trigeminal nerve injury) or central causes (post-stroke). It appears to be much less effective for spinal cord injury pain, post-herpetic neuralgia, or plexus avulsion.

Monsalve reviewed the literature concerning MCS and facial chronic neuropathic pain (an amalgamation of conditions causing neuropathic facial pain) [12]. He found 84% of 100 patients implanted following a trial had at least 40% pain improvement. Lefaucher et al. reported a randomized control trial of MCS for peripheral neuropathic pain where 13 patients had significant reduction in some measurements of pain when the device was ‘on’ compared to ‘off’ [14]. This significance was lost, however, once statistically corrected for multiple comparisons. Nguyen et al. reported a randomized, blinded crossover trial of MCS in 10 patients with neuropathic pain with significant reduction in pain when the device ‘on’ compared to ‘off’ [15]. Of note, there was a poor response in patients with hemibody post-stroke pain and post-herpetic neuralgia. Velasco et al. reported a randomized trial of MCS (some patients were simultaneously reported in the Nguyen paper) and found a similar benefit of MCS in a prospective, blinded trial. Again, several patients (2/5) with post-herpetic neuralgia had no benefit [16]. Velasco et al. also reported that four of five patients with complex regional pain syndrome improved their pain, sensory and sympathetic symptoms in a blinded trial of MCS [17].

These studies had some consistent findings. First, MCS appears to be very effective for deafferentation facial pain. Second, MCS appears to be effective in post-stroke pain. Third, authors often commented (anecdotally) that MCS was not effective for lower limb pain or pain in an area where there was motor weakness.

Two trials have not shown efficacy with MCS but both suffered from methodological flaws. Radic et al. reported that half their twelve patients dropped out of the study and the remaining ‘treatment’ group received what we would consider was subtherapeutic stimulation time (10 min ‘on’ and 120 min ‘off’) [18]. Our preliminary (unpublished) data from an ongoing prospective, randomized trial of MCS with varying time of stimulation has shown that most patients lose the benefit of stimulation once the ‘on’ time is reduced below 50%. Sachs et al. retrospectively reported that only 14% of 14 patients had long term pain relief [19]. Within the cohort, five patients had no motor response to stimulation (a finding some would consider a contraindication to MCS [20]), three had lower limb pain (known to be difficult to treat with MCS [13]) and their stimulation protocol used 30 min ‘wash-out’ (whereas patients blinded to settings usually take three days to notice a difference [2]).

## 5 A comparison of DBS and MCS for neuropathic pain

There is very little data providing a direct comparison between these two techniques. Katayama et al. reported no difference between MCS and thalamic DBS for phantom limb pain [21]. The same group reported the effects of spinal cord stimulation (SCS), thalamic DBS and MCS in 45 patients with post-stroke pain. Satisfactory pain control was obtained more frequently as the stimulation site was moved to higher levels (7% by SCS, 25% by DBS and 48% by MCS) [22]. Nandi et al. reported their experience with treating post-stroke pain with MCS and DBS [23]. Only 1/6 patients had long-term benefit following MCS (although none were trialled) whereas 2/2 patients had longer-term benefit following DBS of the periventricular grey region (4 were trialled). Son et al. recently published a direct comparison of MCS or thalamic DBS in the same eight patients with neuropathic pain [24]. MCS was successful in reducing pain in 6/8 and DBS was successful in 2/8. The improved rate of response and potentially reduced invasiveness led this group to recommend MCS. It is of note that the reported benefits in the individual patients with spinal cord injury were 12.5%, 22%, 37.5% and unavailable. The benefits for the four post stroke patients were all more than 40%.

One obvious difference between the two techniques is that placement of the DBS lead is inherently more invasive than the epidural (or even subdural) MCS electrode.

## 6 Conclusions and direction for future research

Severe neuropathic pain remains a devastating condition. Upwards of 5% of the population may be suffering moderate, medically intractable pain [25]. There are some patients in whom the pain is excruciating and refractory to all medications. For this small cohort, neurosurgery remains their only hope.

Our field needs evidence based guidelines for *each* of the neuropathic pain conditions. In the past, most open label case series have ‘lumped’ many different conditions together and then made generalized statements that a specific operation is effective for neuropathic pain. It has become clear that each of the different pain syndromes may respond differently to neuromodulation. Phantom limb pain, for example, responses more favourably to neuromodulation than brachial plexus avulsion pain. Trigeminal deafferentation pain responds very favourably to neuromodulation whereas facial post-herpetic neuralgia does not. Future trials need to have pure cohorts with only one condition examined at a time. This will require multi-centre collaboration in order to gain the necessary power for analysis. Collaboration will require standardization of technique. This will be easy for the surgery but the post-operative stimulation parameters are equally, if not more, important. Our group has recently published evidence showing that stimulation voltage changes as small as 10% can dramatically alter pain modulation [2]. The very nature of DBS and MCS may allow for blinded evaluation. We recommend future trials incorporate a post-operative phase to optimize stimulation parameters and then a blinded cross-over phase to test the response followed by an open label phase to assess long-term efficacy.

An exciting area of research is the pre-selection of patients using repetitive transcranial magnetic stimulation (rTMS). This non invasive method to stimulate the motor cortex has been shown to predict the beneficial effects of chronic MCS [26], [27]. Finally, the Oxford group continues to lead the field with research into understanding the pain pathways and clinical trials utilizing DBS in new areas — the anterior cingulum [28]. The early results suggest this target may be able to salvage patients who have failed earlier neuromodulation.

Ultimately, we hope there will be a reliable method of neuromodulation (either MCS or DBS) for each of the neuropathic pain conditions with a predictable response rate and minimal complications. The joy and frustration of neurosurgery is captured succinctly in the management of these patients with neuropathic pain.
